# Dissemination of *bla*_NDM–__1_ Gene Among Several *Klebsiella pneumoniae* Sequence Types in Mexico Associated With Horizontal Transfer Mediated by IncF-Like Plasmids

**DOI:** 10.3389/fmicb.2021.611274

**Published:** 2021-03-25

**Authors:** José Eduardo Toledano-Tableros, Catalina Gayosso-Vázquez, Ma Dolores Jarillo-Quijada, José Luis Fernández-Vázquez, Rayo Morfin-Otero, Eduardo Rodríguez-Noriega, Silvia Giono-Cerezo, Gabriel Gutkind, José Di Conza, José Ignacio Santos-Preciado, María Dolores Alcántar-Curiel

**Affiliations:** ^1^Laboratorio de Infectología, Microbiología e Inmunología Clínicas, Unidad de Investigación en Medicina Experimental, Facultad de Medicina, Universidad Nacional Autónoma de México, Ciudad de México, Mexico; ^2^Departamento de Microbiología, Escuela Nacional de Ciencia Biológicas, Instituto Politécnico Nacional, Ciudad de México, Mexico; ^3^Hospital Civil de Guadalajara “Fray Antonio Alcalde” e Instituto de Patología Infecciosa y Experimental, Centro Universitario de Ciencias de la Salud, Universidad de Guadalajara, Guadalajara, Mexico; ^4^Laboratorio de Resistencia Bacteriana, Facultad de Farmacia y Bioquímica de la Universidad de Buenos Aires, Buenos Aires, Argentina

**Keywords:** *Klebsiella pneumoniae*, *bla*_NDM–1_, IncF-like plasmids, carbapenems, MLST, Mexico

## Abstract

Nosocomial infections caused by multidrug-resistant (MDR) *Klebsiella pneumoniae* are a major health problem worldwide. The aim of this study was to describe NDM-1-producing *K. pneumoniae* strains causing bacteremia in a tertiary referral hospital in Mexico. MDR *K. pneumoniae* isolates were screened by polymerase chain reaction for the presence of resistance genes. In resistant isolates, plasmids were identified and conjugation assays were performed. Clonal diversity and the sequence types were determined by pulsed-field gel electrophoresis and multilocus sequence typing. A total of 80 *K. pneumoniae* isolates were collected from patients with bacteremia over a 1-year period. These isolates showed a level of resistance of 59% (47/80) to aztreonam, 56–60% (45–48/80) to cephalosporins, 54% (43/80) to colistin and 12.5% (10/80) to carbapenems. The carbapenem resistant isolates were *bla*_NDM–__1_ carriers and negative for *bla*_KPC_, *bla*_NDM_, *bla*_IMP_, *bla*_VIM_ and *bla*_OXA–__48__–like_ carbapenemases genes. Conjugative plasmids IncFIIA and IncF group with sizes of 82–195 kbp were carriers of *bla*_NDM–__1_, *bla*_CTX–M–__15_, *bla*_TEM–__1_, *aac(6′)-Ib* and/or *aac(3′)-IIa*. Clonal variability and nine different multilocus sequence types were detected (ST661, ST683, ST1395, ST2706, ST252, ST1198, ST690, ST1535, and ST3368) for the first time in the isolates carrying *bla*_NDM–__1_ in Mexico. This study demonstrates that *bla*_NDM–__1_ has remained within this hospital in recent years and suggests that it is currently the most prevalent carbapenemase among *K. pneumoniae* MDR strains causing bacteremia in Mexico. The horizontal transfer of *bla*_NDM–__1_ gene through IncF-like plasmids among different clones demonstrates the dissemination pathway of antimicrobial resistance and underscore the need for strong and urgent joint measures to control the spread of NDM-1 carbapenemase in the hospital.

## Introduction

*Klebsiella pneumoniae* (*K*. *pneumoniae*) is the causative agent of community and hospital acquired infections (Ramirez et al., 2019). In recent years, this bacterium has acquired high resistance to broad-spectrum antibiotics such as β-lactams, aminoglycosides, and quinolones (Ferreira et al., 2019). At the present time, the spread of carbapenemase-producing *K. pneumoniae* is a global public health concern (Villa et al., 2017). *Klebsiella pneumoniae* carbapenemase (KPC) was first reported in North Carolina in 2001. In the last decade it has disseminated globally due to the clonal spread of KPC-producing *K. pneumoniae* and in some countries its nosocomial dissemination has caused outbreaks (Martin et al., 2017). In Mexico, the first report of KPC-3-producing *K. pneumoniae* causing an outbreak was in 2013 (Rodríguez-Zulueta et al., 2013). Two subsequent works have reported the presence of a small number of strains of *K. pneumoniae* producing this carbapenemase (Bocanegra-Ibarias et al., 2017; Aquino-Andrade et al., 2018). One of the carbapenemases initially described in *K. pneumoniae* is New Delhi metallo-β-lactamase 1 (NDM-1), the dissemination of which is mostly hospital associated (Politi et al., 2019). The prevalence of carbapenemases in *K. pneumoniae* has been little studied in Mexico, however, recent research has demonstrated that NDM-1 carbapenemase is more frequent than that of KPC (Rodríguez-Zulueta et al., 2013; Bocanegra-Ibarias et al., 2017; Aquino-Andrade et al., 2018; Alcántar-Curiel et al., 2019b), which has been reported as endemic in the United States, Brazil, Argentina, Colombia and sporadically in Canada (Lee et al., 2016; Hammoudi Halat and Ayoub Moubareck, 2020).

Although *bla*_NDM–__1_ has been found on the bacterial chromosome, the vast majority is carried on plasmids (Wu et al., 2019). Currently there are 20 different incompatibility groups (Inc) of *bla*_NDM–__1_ carrying plasmids in *Enterobacteriaceae*, including IncA/C, IncFIA, IncFIB, IncFII and IncX3 (Wu et al., 2019), indicating the different possibilities of acquisition of *bla*_NDM–__1_ and the horizontal spread between bacteria of the same or different species.

In addition to this phenomenon, some *K. pneumoniae* carbapenemase producers are defined as high-risk clones because of their ability to colonize, spread and persist (Pitout et al., 2015). The multilocus sequence types (ST) ST258, and ST11, both belonging to the clonal complex (CC) 258, are prototypes of an epidemic clone which was identified as early 2000s and are currently spread around the world (Pitout et al., 2015; Lee et al., 2016).

The aims of this study were to investigate antimicrobial resistant genes, the plasmids associated with horizontal gene transfer and to determine the expansion of multilocus sequence types in *K. pneumoniae* causing bacteremia in a tertiary referral hospital in Mexico.

## Materials and Methods

### Bacterial Isolation

Non-duplicate isolates of *K. pneumoniae* were consecutively collected from all blood cultures of patients with nosocomial bacteremia identified from January to December 2017 at Hospital Civil de Guadalajara, an 899-bed tertiary-care teaching hospital in Guadalajara, Jalisco, Mexico. The hospital infrastructure is made up of two buildings, the old, the architecture of the building is mixed, horizontal in its old area and a vertical tower of specialties with ten levels, also there is a building for the Care of Neonates and Women, an outpatient tower, the ophthalmology Unit and a Geriatric Care Unit.

Nosocomial infections were defined according to criteria published by the Centers for Disease Control and by Infectious Diseases Unit physicians (Horan et al., 2008). The isolates were stored in Luria Bertani (LB) broth (Difco, BD Biosciences, Franklin Lakes, NJ, United States) with 20% glycerol (Sigma-Aldrich, St. Louis, MO, United States) at −70°C.

### Antimicrobial Susceptibility Testing

Identification and antimicrobial susceptibility against piperacillin–tazobactam, aztreonam, cefazolin, cefepime, ceftriaxone, ceftolozane–tazobactam, imipenem, meropenem, ciprofloxacin, amikacin, gentamycin, tobramycin, nitrofurantoin, tigecycline and trimethoprim-sulfamethoxazole were performed using the Vitek^®^ 2 system (BioMérieux Durham, NC, United States). Minimal inhibitory concentrations (MIC) of colistin were determined by a microdilution method following the guidelines of the Clinical and Laboratory Standard Institute (CLSI, 2018). The production of extended spectrum β-lactamases (ESBLs) was confirmed phenotypically in all isolates resistant to penicillin/tazobactam and cephalosporins using the agar diffusion method (CLSI, 2018). Metallo-β-lactamases (MBLs) production in carbapenem-resistant isolates was determined by the diffusion test on agar using meropenem and imipenem sensidisks with or without 0.5 M EDTA and in combination with 400 μg/mL of phenylboronic acid for the presumptive identification of carbapenemase KPC (Alcántar-Curiel et al., 2019a).

### Detection of Resistance Genes

Genes that encode antimicrobial resistance were detected by polymerase chain reaction (PCR) assay described previously (Alcántar-Curiel et al., 2019b). The presence of carbapenemase genes *bla*_KPC_, *bla*_NDM_, *bla*_IMP_, *bla*_VIM_ and *bla*_OXA–__48__–like_ was determined by multiplex PCR (Poirel et al., 2011). Endpoint PCR was performed to detect *bla*_TEM_, *bla*_CTX–M_. Additional genes of aminoglycoside modifying enzymes (AMEs) genes *aac(3′)-Ia*, *aac(6′)-IIb*, the methyltransferases genes *rmtB* and *armA* and the colistin resistance *mcr-1* gene (Liu et al., 2016) were included in order to further characterize the strains. The specific oligonucleotides used are described in Supplementary Table 1. The amplified fragments were purified using the Zymogen Purification Kit (Promega) and sequenced (Instituto de Biotecnología, Universidad Nacional Autónoma de México). The sequence analysis was performed with the BioEdit and Kaling bioinformatics tools to subsequently undergo a Basic Local Alignment Search Tool (BLAST) in the National Center for Biotechnology Information database^1^.

### Plasmid Analysis and Conjugation Assays

Plasmids profile was obtained from isolates carrying ESBLs or MBLs according to Eckhardt technique (Eckhardt, 1978). Horizontal transfer of antibiotic resistant was confirmed by bacterial conjugation with *Escherichia coli* J53-2 as the recipient strain using Miller method (Miller, 1992). Transconjugants were selected on McConkey agar supplemented with rifampicin (200 μg/mL) plus ceftazidime (16 μg/mL) and plus meropenem (16 μg/mL) for isolates that were carrying carbapenemases genes and tested for antimicrobial susceptibility. Successful conjugation was confirmed by specific PCR amplification and the electrophoretic pattern of the conjugated plasmids was obtained. The bacterial artificial chromosomes (BACs) of 67, 86, 101, 122, 145, and 195 kb were used as a molecular weight markers (González et al., 2006). Plasmids of transconjugant strains were purified using the QIAGEN Plasmid Midi Kit (Qiagen, Hilden, Germany), following the manufacturer’s specifications. Plasmids diversity was determined by restriction fragment length polymorphism (RFLP) (Ho et al., 2012) with *Eco*RI and *Hin*dIII (Invitrogen) restriction enzymes following the manufacturer’s specifications. Finally, the groups Inc of conjugative plasmids were determined by PCR-based replicon typing (Carattoli et al., 2005).

### Genotyping by Pulsed-Field Gel Electrophoresis

Clonality among all of the isolates was determined by pulsed-field gel electrophoresis (PFGE) (Alcántar-Curiel et al., 2019a). Chromosomal DNA of each isolate was prepared as described previously (Miranda et al., 1996) and macrorestricted with the restriction endonuclease *Xba*I (New England Biolabs, Beverly, MA, United States). Restriction fragments were resolved in a Gene Path System (BioRad^®^, Hercules, CA, United States). The classification of the isolates in clones was based on Tenover criteria (Tenover et al., 1995). The percentage of similarity profile was calculated using the Dice coefficient. Isolates with a Dice similarity coefficient >85% were considered as members of the same clone (Alcántar-Curiel et al., 2019a).

### Multilocus Sequence Typing

To determine the sequence type (ST) of *K. pneumoniae* isolates harboring *bla*_NDM–__1_, MLST was performed according to the Pasteur scheme (Diancourt et al., 2005). The housekeeping genes (*gapA*, *infB*, *mdh*, *pgi*, *phoE*, *rpoB*, *tonB*) were amplified, sequenced, and analyzed with the MLST database of the Pasteur Institute to identify allelic profile. In order to identify the clonal complex (CC) and visualize evolutionary relationships among isolates carrying *bla*_NDM–__1_, we used Phyloviz 2.0 program that generates eBURST and neighbor-joining diagram (Sepp et al., 2019).

## Results

### Clinical Isolates and Antibiotic Susceptibility Pattern

A total of 80 isolates of *K. pneumoniae* causing bacteremia were collected over the course of 1 year at the Hospital Civil de Guadalajara. These isolates representing 8% of the total documented bacteremias. *K. pneumoniae* were more frequently derived in patients from surgical ward (32.5%) and medicine ward (31.2%) (Table 1 and Supplementary Data Sheet 1). The isolates were resistant to penicillin/tazobactam 26% (21/80), aztreonam 59% (47/80), cefepime 56% (45/80), ceftriaxone 60% (48/80), imipenem and meropenem 12.5% (10/80), ciprofloxacin 18% (14/80), tobramycin 50% (40/80), gentamicin 55% (44/80), nitrofurantoin 16% (13/80), tigecycline 5% (4/80), trimethoprim-sulfamethoxazole 56% (45/80) and colistin 53% (42/80) (Table 2).

**TABLE 1:** Frequency of *bla*_NDM–__1_-producing *Klebsiella pneumoniae* isolates in the different hospital setting.

Hospital wards	Area	No. isolates (%)
Surgical		26 (32.5)
Neurology (NEU)	Old	13 (16)
General surgery (GES)	Old	10 (12.5)
Plastic surgery (PSU)	Old	1 (3.8)
Oral and maxillofacial surgery (OMS)	Old	1 (1.3)
Otorhinolaryngology (OTO)	Old	1 (3.8)
Medicine		25 (31.2)
Internal medicine (IME)	Old	7 (8.4)
Cardiology (CAR)	Old	5 (6.3)
Nephrology (NEP)	Tower 5 floor	5 (6.3)
Haematology (HEM)	Tower 9 floor	3 (3.8)
Infectious diseases unit (IDU)	Tower 7 floor	3 (3.8)
Gastroenterology (GAS)	Tower 6 floor	1 (1.3)
HIV/AIDS Unit (HIV)		1 (1.3)
Pediatric intensive care unit		20 (25)
Neonatal intensive care unit (NICU)	Newborn unit	13 (16.2)
Pediatric intensive care unit (PICU)	Tower 1 floor	7 (8.7)
Intensive Care Unit (ICU)	Tower 1 floor	9 (11.2)
Total		80 (100)

**TABLE 2:** Minimum inhibitory concentration data and antimicrobial susceptibility of 80 *Klebsiella pneumoniae* isolates from January to December 2017 at Hospital Civil de Guadalajara.

Drug class	Antimicrobial agent	MIC* (μg/mL)		Antimicrobial susceptibility (%)		
		MIC_90_	MIC_50_	Susceptible	Intermediate	Resistant
β-lactam combination agents	Piperacillin/Tazobactam	128	16	65	9	26
Monobactam	Aztreonam	64	2	36	5	59
Cephems	Cefazolin	64	8	41	0	59
	Cefepime	64	2	44	0	56
	ceftriaxone	64	32	40	0	60
β-lactam combination agents	Ceftolozane/Tazobactam**	N/A	N/A	87	0	13
Carbapenems	Imipenem	8	0.06	87	0	13
	Meropenem	4	0.03	87	0	13
Fluoroquinolones	Ciprofloxacin	4	1	68	14	18
Aminoglycosides	Amikacin	128	8	56	0	24
	Gentamicin	128	32	45	0	55
	Tobramycin	32	1	50	0	50
Nitrofurans	Nitrofurantoin	128	64	26	58	16
Glycylcycline	Tigecycline***	2	1	91	4	5
Folate pathway antagonists	Trimethoprim/Sulfamethoxazole	16/304	16/304	43	1	56
Lipopeptides	Colistin***	32	4	47	0	54

*Susceptibility breakpoint categories were derived from CLSI (2018). **Susceptibility was determined by agar disk diffusion method. ***Susceptibility categorization was determined according to the EUCAST criteria. N/A, Not applicable.

### Antibiotic Resistance Enzymes

A total of 10 strains resistant to all β-lactams including the two carbapenems were detected. These strains were MBL producers and carried the *bla*_NDM–__1_ carbapenemase gene (Supplementary Data Sheet 1). Furthermore, three of these strains carried the *bla*_TEM–__1_ penicillinase gene and four strains *bla*_TEM–__1_ and *bla*_CTX–M–__15_ (Supplementary Figure 1 and Supplementary Data Sheet 1). Regarding to the 37 strains resistant to β-lactams but susceptible to carbapenems, 89% (33/37) were ESBL producers; 32 strains carried *bla*_TEM–__1_ and *bla*_CTX–M–__15_ genes and one strain carried only *bla*_CTX–M–__15_ (Supplementary Figure 1 and Supplementary Data Sheet 1).

In relation to the 55 isolates resistant to aminoglycosides tested, 56% (31/55) were carriers of *aac(3′)-IIa* and *aac(6′)-IIb* AME genes. AMEs genes were not detected in five isolates resistant only to amikacin and six isolates resistant to amikacin and gentamicin. *aac(6′)-Ib* was associated with resistance to tobramycin, while *aac(3′)-IIa* was associated with resistance to gentamicin (Supplementary Table 2). Regarding the *mcr-1* gene was not detected in 43 colistin-resistant isolates examined in this study.

### Plasmid Analysis

Conjugation experiments in ten isolates *bla*_NDM–__1_ carriers showed that five transconjugants acquired the *bla*_NDM–__1_ gene (Table 3). Plasmid analysis indicated that one transconjugant harbored the *bla*_NDM–__1_ gene on a ∼82 kbp plasmid. The other four transconjugants harbored the *bla*_NDM–__1_ gene on a ∼195 kbp plasmid, only two of these plasmids were carriers of a single *bla*_NDM–__1_ resistance gene, the other plasmid was a carrier of both *bla*_NDM–__1_ and *bla*_TEM–__1_ genes and the fourth plasmid was a carrier of *bla*_NDM–__1_, *bla*_TEM–__1_, *bla*_CTX–M–__15_ and *aac(3′)-IIa* and *aac(6′)-Ib*. All the five transconjugants were resistant to all β-lactams and aminoglycosides with the exception of one. Plasmid replicon typing showed that four of the conjugative plasmids belonged to the IncFIIA and one to the IncF group.

**TABLE 3:** Antimicrobial susceptibility of 5 *K. pneumoniae* carriers *bla*_NDM–__1_ and their transconjugants.

Isolate	Multiresistant pattern	Resistant genes	Plasmids pattern		Transconjugant	Acquired multi-resistance profile	Acquired resistant genes	Conjugative plasmid size (kbp)
			No.	Size (kbp)				
12-Kpn	PTZ, AZT, CFZ, FEP, CRO, CAZ, C/T, IMI, MEM, CIP, TOB, NIT, SXT, COL	*bla*_NDM–__1_, *bla*_CTX–M–__15_, *bla*_TEM–__1_	3	>195 195 82	12-Tc	PTZ, AZT, CFZ, FEP, CRO, CAZ, C/T, IMI, MEM	*bla*_NDM–__1_ *bla*_CTX–M–__15_	82
18-Kpn	PTZ, AZT, CFZ, FEP, CRO, CAZ, C/T, IMI, MEM, AMK, GEN, TOB, NIT, COL	*bla*_NDM–__1_, *bla*_TEM–__1_	1	195	18-Tc	PTZ, AZT, CFZ, FEP, CRO, CAZ, C/T, IMI, MEM, AMK, GEN, TOB	*bla*_NDM–__1_, *bla*_TEM–__1_	195
19-Kpn	PTZ, AZT, CFZ, FEP, CRO, CAZ, C/T, IMI, MEM, GEN, TOB, NIT, CIP, COL	*bla*_NDM–__1_, *bla*_CTX–M–__15_, *bla*_TEM–__1_, *aac(6′)-Ib, aac(3′)-IIa*	2	>195 195	19-Tc	PTZ, AZT, CFZ, FEP, CRO, CAZ, C/T, IMI, MEM, GEN, TOB, CIP	*bla*_NDM–__1_, *bla*_CTX–M–__15_, *bla*_TEM–__1_, *aac(6′)-Ib, aac(3′)-IIa*	195
40-Kpn	PTZ, AZT, CFZ, FEP, CRO, CAZ, C/T, IMI, MEM, AMK, GEN, TOB, NIT, TGC, SXT	*bla*_NDM–__1_, *bla*_TEM–__1_	1	195	40-Tc	PTZ, AZT, CFZ, FEP, CRO, CAZ, C/T, IMI, MEM, AMK, GEN, TOB	*bla*_NDM–__1_	195
41-Kpn	PTZ, AZT, CFZ, FEP, CRO, CAZ, C/T, IMI, MEM, AMK, GEN, TOB, NIT, TGC, SXT, COL	*bla*_NDM–__1_, *bla*_TEM–__1_	1	195	41-Tc	PTZ, AZT, CFZ, FEP, CRO, CAZ, C/T, IMI, MEM, AMK, GEN, TOB	*bla*_NDM–__1_	195

*PTZ, piperacillin-tazobactam; AZT, Aztreonam; CAZ, ceftazidime; CRO, ceftriaxone; C/T, ceftolozane-tazobactam; CFZ, cefazolin; FEP, cefepime; IMP, imipenem; MEM, meropenem; AMK, amikacin; GEN, gentamicin; TOB, tobramycin; CIP, ciprofloxacin; NIT, nitrofurantoin; TGC, tigecycline; SXT, trimethoprim-sulfamethoxazole; COL, colistin.*

With respect to thirty-three *bla*_TEM–__1_ and *bla*_CTX–M–__15_ carriers, 26 transconjugants were obtained (Supplementary Table 3), of which 13 harbored a >195 kbp plasmid, 12 carried a ∼195 kbp plasmid and only one acquired a ∼67 kbp plasmid. From the total 26 conjugative plasmids, 25 of them harbored the *bla*_CTX–M–15_, *bla*_TEM–1_, *aac*(3′)-*IIa* and *aac*(6′)-*Ib*, and only one of them harbored the *bla*_CTX–M–15_, *bla*_TEM–1_ and *aac*(6′)-*IIa*. Twenty-five of these plasmids belonged to the IncF group and only one plasmid to the IncFIIA group.

**FIGURE 1:**
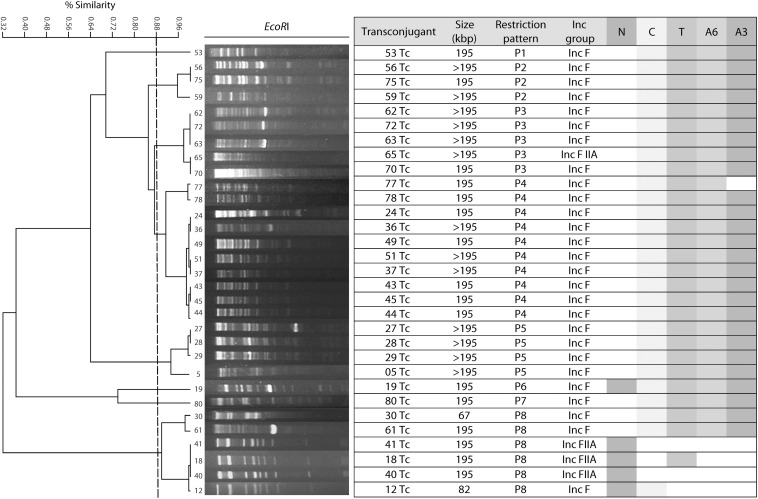
Restriction fragment length polymorphism analysis of conjugative plasmids carrying antimicrobial resistance genes. N: *bla*_NDM–1_, C: *bla*_CTX–M–15_, T: *bla*_TEM–1_, A6: *aac*(6′)-*Ib*, A3: *aac*(3′)-*IIa*.

Fragment length polymorphism (RFLP) analysis of the conjugative plasmids carrying both *bla*_NDM–__1_ and *bla*_CTX–M–__15_ showed an average similarity of 88% (Figure 1). The five plasmids carriers *bla*_NDM–__1_ revealed two different restriction profiles (P), four of them belonged to P8. The 26 plasmids carriers of *bla*_TEM–__1_ and *bla*_CTX–M–__15_ belonged to eight different restriction profiles.

### Clonality Analysis

Pulsed-field gel electrophoresis (PFGE) analysis was conducted with an average similarity of 66%. Sixty-nine different clones were detected among the 80 isolates, which showed clonal heterogeneity (data not shown). Clone 26 was the most prevalent with three isolates collected in May and June, all carriers of *bla*_CTX–M–__15_, *bla*_TEM–__1_, *aac(3′)-Ia* and *aac(6′)-IIb*. Clones 6, 15, 34, 35, 37, 39, 42, and 47 had two isolates each, while the rest of the isolates belonged to different clones (Supplementary Data Sheet 1). The 43 strains of *K. pneumoniae* carrying resistance genes belonged to 38 different clones, the 10 isolates carriers of *bla*_NDM–__1_ belonged to different clones (Supplementary Figure 1).

### MLST Analysis

The analysis demonstrated nine different STs among ten isolates carriers of *bla*_NDM–__1_ gene_;_ ST661, ST683 belonged to CC258, ST1395, ST2706, ST252, ST1198, ST690, ST1535, and ST3368 (Supplementary Figure 2). Isolates 07-KP-17, and 11-KP-17 belonged to ST661 which corresponds to the founder member of CC661, these isolates were recovered in February and March 2017 respectively.

**FIGURE 2:**
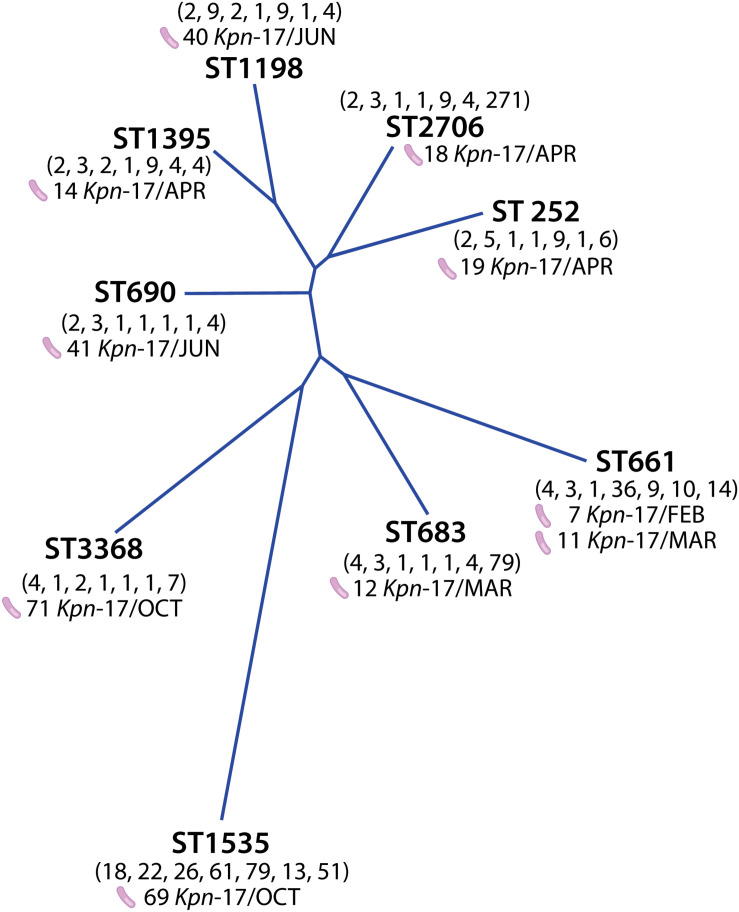
Phylogenetic analysis of STs obtained for *bla*_NDM–__1_-producing *K. pneumoniae* strains using the neighbor-joining method.

Phylogenetic analysis using the neighbor-joining method detected the genetic distance between the 10 isolates carriers of *bla*_NDM–__1_ gene (Figure 2). Isolates recovered in February and March as well as the isolates recovered between April and June were significantly associated, while the October isolates including the 69-KP-17 isolate were not.

## Discussion

*Klebsiella pneumoniae* is included in the global priority list of antibiotic resistant bacteria, and there is a need for enhanced *K. pneumoniae* surveillance to rapidly identify and monitor convergent strains and/or plasmids (Wyres et al., 2020). The infections caused by *K. pneumoniae* during the present study period were 8%, lower than those reported for developing countries ranging from 16 to 28% (Khaertynov et al., 2018).

The rate of antimicrobial resistance observed in the isolates from our study was lower than the rate from the isolates that caused nosocomial outbreaks previously reported in the same hospital (Bocanegra-Ibarias et al., 2017). While this was a short study period, our findings are consistent with the high rate of resistance reported in Asia where they detected 60–75.8% to cephalosporins, 47–65.6% to imipenem and 40.8–76% to amikacin (Effah et al., 2020). Detection of 54% colistin resistance in this study is undoubtedly our biggest concern, a significant increase compared to the 4.7% reported 2 years earlier in this hospital (Bocanegra-Ibarias et al., 2017). This may be due to the increased use of colistin for the treatment of bacteremia caused by carbapenemase-producing isolates in hospital and because the isolates from the previous study were mostly derived from a nosocomial outbreak (Bocanegra-Ibarias et al., 2017).

The spread of NDM-producing bacteria and their association with nosocomial outbreaks is of concern worldwide (Dortet et al., 2014). The first report of NDM in *K. pneumoniae* was described in Mexico in 2014 (Barrios et al., 2014). The detection of *bla*_NDM–__1_ in ten isolates included in this study, demonstrates the spread and persistence of this carbapenemase among *K. pneumoniae* isolates for at least two consecutive years at Hospital Civil de Guadalajara (Bocanegra-Ibarias et al., 2017). Additionally, the co-transfer of *bla*_NDM–__1_ and AMEs genes together with fluoroquinolone resistance in 19-Kpn isolate (Table 3) demonstrates the concurrence of these genes, which represents a major challenge in the treatment of patients (Mitra et al., 2019).

It has been documented that in *K. pneumoniae* these resistance genes are encoded in small 25 kbp conjugative plasmids or smaller (Ramirez et al., 2019), which contrasts with the >195 kbp conjugative plasmids identified in this study (Figure 1). RFLP analysis showed that isolates in our study harbor different types of plasmids carrying *bla*_NDM–__1_, ESBLs and AMEs, suggesting that genetic rearrangements occurred at the plasmid level during this period of study. Furthermore, similar plasmids were detected among different clones, which indicates that the transfer of genes is common among bacteria allowing the spread of resistance genes in the hospital environment.

The *bla*_NDM–__1_ gene has been identified in plasmids from different replicon types, in this study the plasmid carrying *bla*_NDM–__1_ gene belongs to the IncF and IncFIIA subgroups; which are different from the IncFIIk and IncFIIy subgroups previously reported in this hospital (Bocanegra-Ibarias et al., 2017) and the IncFIA reported in isolates in Mexico City (Alcántar-Curiel et al., 2019a).

The prevalence of plasmid replicons is led by the IncX group, while the IncF group ranks third, worldwide (Partridge et al., 2018; Wu et al., 2019). However, in Latin America, particularly in Mexico, Brazil, and Colombia, the IncF group seems be the most prevalent (Torres-González et al., 2015; Bocanegra-Ibarias et al., 2017; Alcántar-Curiel et al., 2019b; Wu et al., 2019).

In this study, gentamicin and tobramycin resistance was associated with the production of AAC(3′)-IIa and AAC(6′)-Ib, but not for amikacin resistance since the resistant isolates did not carry these genes studied (Supplementary Table 2). The five transconjugants of the isolates that produced ESBLs and were resistant to the 3 aminoglycosides acquired both AMEs but only had resistance to gentamicin and tobramycin (Table 2), suggests that amikacin resistance may be due to another AME or another resistance mechanism not encoded in plasmids. Because none of the studied AMEs were detected in transconjugants carriers *bla*_NDM–__1_ that acquired resistance to aminoglycosides, we consider that these isolates may carry others AMEs that were not investigated.

One of the most prevalent genes in *Enterobacteriaceae*, *Pseudomonadales* and *Vibrionaceace* worldwide is *aac(6′)-Ib* (Ramirez and Tolmasky, 2017; Fernández-Martínez et al., 2018; Galani et al., 2019), which is mostly associated with amikacin and gentamicin resistance (Ramirez and Tolmasky, 2017; Ramirez et al., 2019) and frequently encoded in plasmids and coexisting with ESBLs such as CTX-M. Our results are in agreement with these data with the exception that *aac(6′)-Ib* seems to be associated with tobramycin resistance since it was detected in isolates resistant only to this antibiotic. The observation that *aac(3′)-IIa* is more frequent in *Enterobacteriaceae* and is associated with gentamicin and tobramycin resistance (Fernández-Martínez et al., 2018) is in agreement with our results.

Colistin resistance was not transferred by conjugation and this was supported by the fact that none of the isolates carried the mcr-1 gene, suggesting that resistance may be due to be associated with chromosomal mutations that are directly involved in LPS modifications such alteration in the MgrB gene, a very common colistin resistance mechanism in *K. pneumoniae* from the clinical setting (Luo et al., 2017).

Throughout our study, it was interesting to find that isolates showed a wide clonal diversity, including the carbapenem-resistant isolates which carried the *bla*_NDM–__1_ gene that has been frequently associated with outbreaks by *K. pneumoniae* (Dortet et al., 2014). However, the plasmids *bla*_NDM–__1_ carriers and other resistance genes were similar, their detection in different clones partially explains their dissemination in different clones, coinciding with previously report of *bla*_NDM–__1_ carriers *Enterobacteriaceae* in this hospital (Bocanegra-Ibarias et al., 2017).

The 9 STs detected in the ten *K. pneumoniae* carriers of *bla*_NDM–__1_ gene have not been previously described in Mexico (Barrios et al., 2014; Torres-González et al., 2015; Bocanegra-Ibarias et al., 2017, 2019; Garza-Ramos et al., 2018). However, the isolate belonging to the ST683 is related to clonal complex 258, an epidemic clone with a global expansion. This clone is prevalent in Argentina and includes multi-drug resistant microorganisms that are KPC-producing and have been associated with high mortality rates (Cejas et al., 2019). Finally, three STs detected in this study (ST661, ST690, ST252) have been previously reported in other regions of the world although none of these *K. pneumoniae* strains carriers the *bla*_NDM–__1_ gene (Coelho et al., 2012; Papagiannitsis et al., 2015; Martin et al., 2017; Fu et al., 2018; Marques et al., 2019; Piazza et al., 2019; Sghaier et al., 2019; Mori et al., 2020).

## Conclusion

This study shows the prevalence of *K. pneumoniae* MDR isolates causing bacteremia in a tertiary referral hospital in Mexico. The carbapenem-resistant isolates were carriers of the *bla*_NDM–__1_ gene harbored in similar IncF-like plasmids among clones with different STs, which supports their nosocomial dissemination and persistence in different plasmids which can be associated with genetic rearrangements that might be in favor the microevolution of this nosocomial pathogen. These results underscore the importance of maintaining microbiological and epidemiological surveillance actions to detect and prevent the spread of MDR bacteria.

## Data Availability Statement

The original contributions presented in the study are included in the article/Supplementary Material, further inquiries can be directed to the corresponding author/s.

## Ethics Statement

This study was evaluated and approved by the Institutional Research and Ethics Committee of the Hospital Civil de Guadalajara project number HCG/CEI-009316; 27 January 2016. The study does not involve humans it is an *in vitro* study. Written informed consent was not required for this study according to the institutional ethical, biosecurity and investigation committees because the Hospital Clinical Laboratory provided every bacterial isolates included in this study.

## Author Contributions

JET-T conceived and designed the study and performed the experiments, analyzed the data, and wrote and edited the manuscript. CG-V, MDJ-Q, JLF-V, and JDC performed the experiments, analyzed the data, and revised the manuscript. RM-O, ER-N, SG-C, GG, and JS-P analyzed the data and revised the manuscript. MA-C conceived, designed, and supervised the study, analyzed the data, and wrote and edited the manuscript. All authors contributed to the article and approved the submitted version.

## Conflict of Interest

The authors declare that the research was conducted in the absence of any commercial or financial relationships that could be construed as a potential conflict of interest.

## Footnotes

1 www.ncbi.nlm.nih.gov/blast/

## Supplementary Material

Supplementary Figure 1 Genetic relationship and molecular characteristics of the 43 strains of *K. pneumoniae* carrying *bla*_NDM–1_ and other resistance genes.

Supplementary Figure 2 Diagram of the different clonal complexes (CC) and the multilocus sequence types (STs) identified by eBURST in *bla*_NDM–1_-producing *K. pneumoniae* isolates. The ST (red circles) and the CC (green circles) obtained for each strain 2B.
