# Supplementary figures and images for: Dissemination of blaNDM–1 Gene Among Several Klebsiella pneumoniae Sequence Types in Mexico Associated With Horizontal Transfer Mediated by IncF-Like Plasmids
Source: Front Microbiol. 2021 Mar 25;12:611274. doi: 10.3389/fmicb.2021.611274 (PMC8027308; doi:10.3389/fmicb.2021.611274)

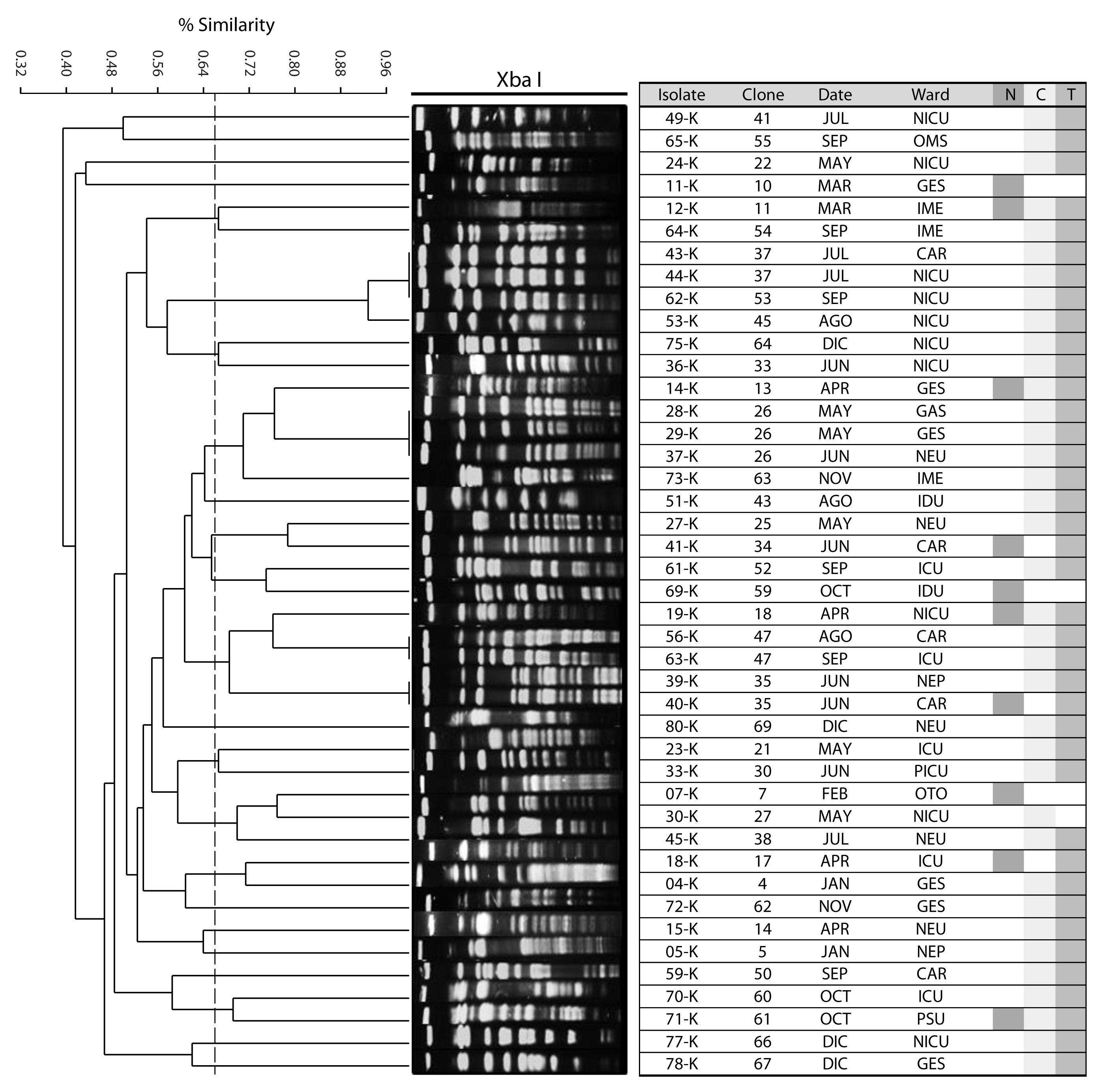

Supplement: Supplementary Figure 1 — Genetic relationship and molecular characteristics of the 43 strains of K. pneumoniae carrying blaNDM–1 and other resistance genes. [file Image_1.TIF]

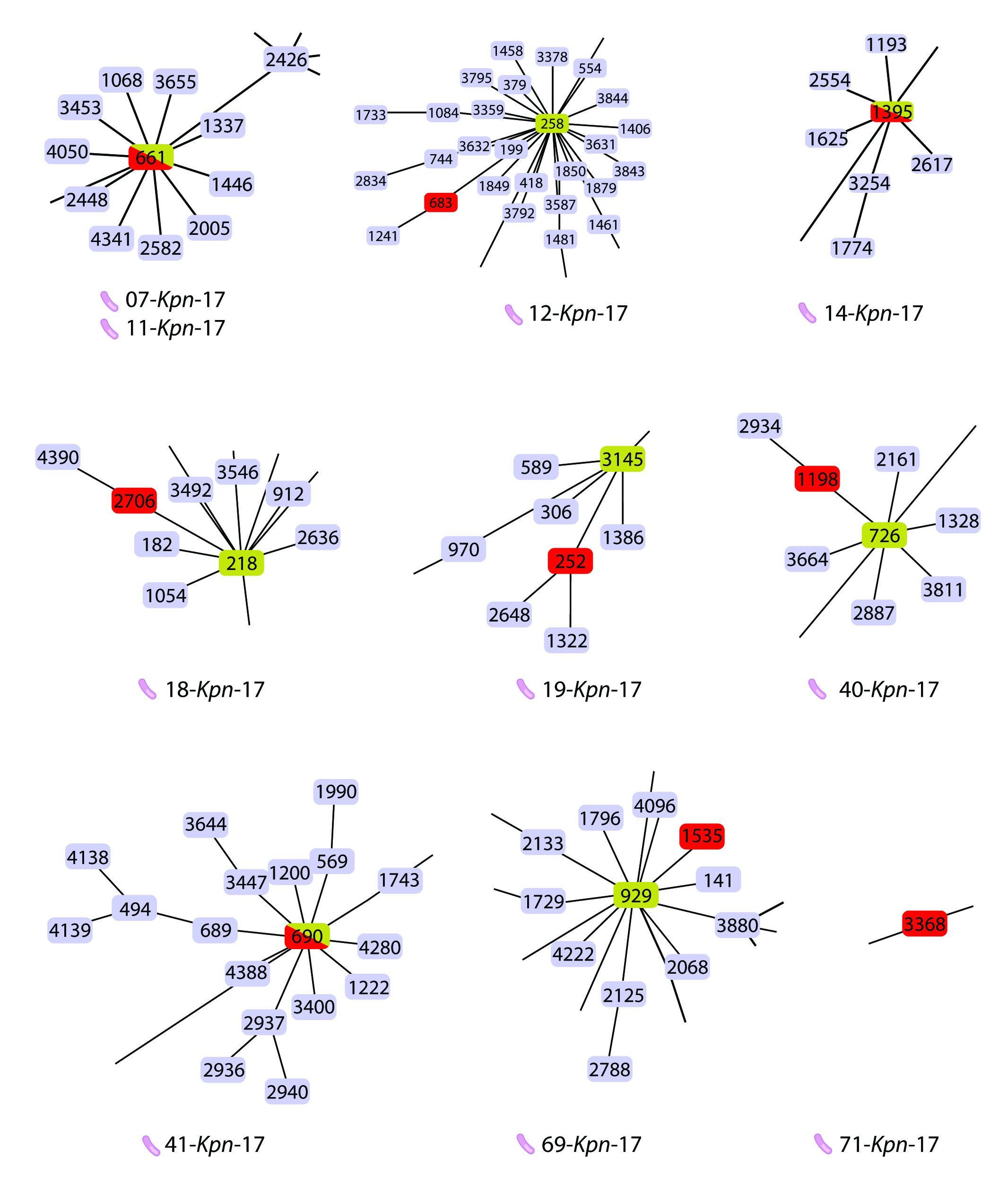

Supplement: Supplementary Figure 2 — Diagram of the different clonal complexes (CC) and the multilocus sequence types (STs) identified by eBURST in blaNDM–1-producing K. pneumoniae isolates. The ST (red circles) and the CC (green circles) obtained for each strain 2B. [file Image_2.TIF]
