# Supplementary material for: Dissemination of blaNDM–1 Gene Among Several Klebsiella pneumoniae Sequence Types in Mexico Associated With Horizontal Transfer Mediated by IncF-Like Plasmids
Source: Front Microbiol. 2021 Mar 25;12:611274. doi: 10.3389/fmicb.2021.611274 (PMC8027308; doi:10.3389/fmicb.2021.611274)
Supplement: Supplementary file 4 [file Table_1.docx]

Supplementary Table 1. Oligonucleotides used in this study to detect resistance genes by PCR.

| Gen | Sequence 5´🡪 3´ | Amplicon size (kbp) | Reference |
| --- | --- | --- | --- |
| *aac(6´)-Ib* | Fw: TTGCGATGCTCTATGAGTGGCTA | 482 | (Fernández-Martínez et al., 2018) |
|  | Rv: CTCGAATGCCTGGCGTGTTT |  |  |
| *aac(3´)-IIa* | Fw: GGCAATAACGGAGGCGCTTCAAAA | 563 |  |
|  | Rv: TTCCAGGCATCGGCATCTCATACG |  |  |
| *armA* | Fw: CCGAAATGACAGTTCCTATC | 846 | (Hu et al., 2013) |
|  | Rv: GAAAATGAGTGCCTTGGAGG |  |  |
| *rmtB* | Fw: ATGAACATCAACGATGCCCTC | 769 |  |
|  | Rv: CCTTCTGATTGGCTTATCCA |  |  |
| *bla*_NDM_ | Fw: GGTTTGGCGATCTGGTTTTC | 621 | (Poirel et al., 2011) |
|  | Rv: CGGAATGGCTCATCACGATC |  |  |
| *bla*_KPC_ | Fw: CGTCTAGTTCTGCTGTCTTG | 798 |  |
|  | Rv: CTTGTCATCCTTGTTAGGCG |  |  |
| *bla*_IMP_ | Fw: GGAATAGAGTGGCTTAAYTCTC | 232 |  |
|  | Rv: GGTTTAAYAAAACAACCACC |  |  |
| *bla*_VIM_ | Fw: GATGGTGTTTGGTCGCATA | 390 |  |
|  | Rv: CGAATGCGCAGCACCAG |  |  |
| *bla*_OXA-48-like_ | Fw: GCGTGGTTAAGGATGAACAC | 438 |  |
|  | Rv: CATCAAGTTCAACCCAACCG |  |  |
| *bla*_TEM_ | Fw: ATGAGTATTCAACATTTTCG | 861 | (Celenza et al., 2006) |
|  | Rv: TTACCAATGCTTAATCAGTGAG |  |  |
| *bla*_CTX-M_ | Fw: CGCTTTGCGATGTGCAG | 550 | (Dutour et al., 2002) |
|  | Rv: ACCGCGATATCGTTGGT |  |  |
| *cmr-1* | CLR5-F (5ʹ-CGGTCAGTCCGTTTGTTC-3ʹ) | 309 | (Liu et al., 2016) |
|  | CLR5-R (5ʹ-CTTGGTCGGTCTGTA GGG-3ʹ) |  |  |
