# Supplementary material for: Dissemination of blaNDM–1 Gene Among Several Klebsiella pneumoniae Sequence Types in Mexico Associated With Horizontal Transfer Mediated by IncF-Like Plasmids
Source: Front Microbiol. 2021 Mar 25;12:611274. doi: 10.3389/fmicb.2021.611274 (PMC8027308; doi:10.3389/fmicb.2021.611274)
Supplement: Supplementary file 5 [file Table_2.docx]

| Aminoglycoside resistance phenotype | | Aminoglycoside-modifying-enzyme gene | | |
| --- | --- | --- | --- | --- |
| Antibiotic | Isolates (n=80) | *aac(3´)-IIa* +  *aac(6´)-Ib* | *aac(6´)-Ib* | *aac(3´)-IIa* |
| AMK, GEN, TOB | 13 (15%) | 8 (15%) | 1 (2%) | 1 (2%) |
| AMK, GEN | 6 (8%) | 0 | 0 | 0 |
| AMK | 5 (6%) | 0 | 0 | 0 |
| GEN, TOB | 22 (28%) | 19 (35%) | 1 (2%) | 2 (4%) |
| GEN | 3 (4%) | 2(4%) | 0 | 1 (2%) |
| TOB | 6 (8%) | 2 (4%) | 4 (7%) | 0 |
| Total resistant isolates | 55 (69 %) | 31 (56%) | 6 (11%) | 4 (7%) |

Supplementary Table 2. Prevalence of aminoglycoside resistance K. pneumoniae and aminoacyltransferase genes associated with aminoglycoside resistance.

AMK: amikacin, GEN: gentamicin, TOB; tobramycin.
