# Supplementary material for: Dissemination of blaNDM–1 Gene Among Several Klebsiella pneumoniae Sequence Types in Mexico Associated With Horizontal Transfer Mediated by IncF-Like Plasmids
Source: Front Microbiol. 2021 Mar 25;12:611274. doi: 10.3389/fmicb.2021.611274 (PMC8027308; doi:10.3389/fmicb.2021.611274)
Supplement: Supplementary file 6 [file Table_3.docx]

| Supplementary Table 3. Antimicrobial susceptibility of 26 *K. pneumoniae* carriers *bla*_CTX-M-15_ and their transconjugants*.* | | | | | | | | |
| --- | --- | --- | --- | --- | --- | --- | --- | --- |
| Isolate | MDR profile | Resistant genes | Plasmids | | Transconjugant | Acquired multi-resistance profile | Acquired resistant genes | Conjugative plasmid  size (kpb) |
|  |  |  | No | Size (kpb) |  |  |  |  |
| 05-Kpn-17 | PTZ, AZT, CFZ, FEP, CRO, CAZ, GEN, TOB, NIT, SXT, COL | *bla*_CTX-M-15_, *bla*_TEM-1,_ *aac(6’)-Ib, aac(3’)-IIa* | 2 | >195  67 | 05-Tc | PTZ, AZT, CFZ, FEP, CRO, CAZ, C/T, GEN, TOB | *bla*_CTX-M-15_, *bla*_TEM-1,_ *aac(6’)-Ib, aac(3’)-IIa* | >195 |
| 24-Kpn-17 | PTZ, AZT, CFZ, FEP, CRO, CAZ, AMK, GEN, TOB, NIT, SXT | *bla*_CTX-M-15_, *bla*_TEM-1,_ *aac(6’)-Ib, aac(3’)-IIa* | 1 | 195 | 24-Tc | PTZ, AZT, CFZ, FEP, CRO, CAZ, GEN, TOB | *bla*_CTX-M-15_, *bla*_TEM-1,_ *aac(6’)-Ib, aac(3’)-IIa* | 195 |
| 027-Kpn-17 | AZT, CFZ, FEP, CRO, CAZ, CIP, AMK, GEN, TOB, NIT, SXT, COL | *bla*_CTX-M-15_, *bla*_TEM-1,_ *aac(6’)-Ib, aac(3’)-IIa* | 1 | >195 | 27-Tc | AZT, CFZ, FEP, CRO, CAZ, CIP, GEN, TOB | *bla*_CTX-M-15_, *bla*_TEM-1,_ *aac(6’)-Ib, aac(3’)-IIa* | >195 |
| 28-Kpn-17 | AZT, CFZ, FEP, CRO, CAZ, AMK, GEN, TOB, SXT, COL | *bla*_CTX-M-15_, *bla*_TEM-1,_ *aac(6’)-Ib, aac(3’)-IIa* | 1 | >195 | 28 -Tc | AZT, CFZ, FEP, CRO, CAZ, GEN, TOB | *bla*_CTX-M-15_, *bla*_TEM-1,_ *aac(6’)-Ib, aac(3’)-IIa* | >195 |
| 29-Kpn-17 | AZT, CFZ, FEP, CRO, CAZ, GEN, TOB, SXT | *bla*_CTX-M-15_, *bla*_TEM-1,_ *aac(6’)-Ib, aac(3’)-IIa* | 1 | >195 | 29 -Tc | AZT, CFZ, FEP, CRO, CAZ, GEN, TOB | *bla*_CTX-M-15_, *bla*_TEM-1,_ *aac(6’)-Ib, aac(3’)-IIa* | >195 |
| 30-Kpn-17 | AZT, CFZ, FEP, CRO, CAZ, COL | *bla*_CTX-M-15_, | 2 | >195  67 | 30 -Tc | AZT, CFZ, FEP, CRO, CAZ | *bla*_CTX-M-15_, | 67 |
| 36-Kpn-17 | AZT, CFZ, FEP, CRO, CAZ, GEN, TOB, CIP, NIT, TGC, SXT | *bla*_CTX-M-15_, *bla*_TEM-1,_ *aac(6’)-Ib, aac(3’)-IIa* | 1 | >195 | 36 -Tc | AZT, CFZ, FEP, CRO, CAZ, GEN, TOB, | *bla*_CTX-M-15_, *bla*_TEM-1,_ *aac(6’)-Ib, aac(3’)-IIa* | >195 |
| 37-Kpn-17 | PTZ, AZT, CFZ, FEP, CRO, CAZ, GEN, TOB, CIP, NIT, TGC, SXT | *bla*_CTX-M-15_, *bla*_TEM-1,_ *aac(6’)-Ib, aac(3’)-IIa* | 1 | >195 | 37-Tc | PTZ, AZT, CFZ, FEP, CRO, CAZ, GEN, TOB | *bla*_CTX-M-15_, *bla*_TEM-1,_ *aac(6’)-Ib, aac(3’)-IIa* | >195 |
| 43-Kpn-17 | AZT, CFZ, FEP, CRO, CAZ, GEN, TOB, CIP, NIT, SXT | *bla*_CTX-M-15_, *bla*_TEM-1,_ *aac(6’)-Ib, aac(3’)-IIa* | 1 | 195 | 43-Tc | PTZ, AZT, CFZ, FEP, CRO, CAZ, GEN, TOB | *bla*_CTX-M-15_, *bla*_TEM-1,_ *aac(6’)-Ib, aac(3’)-IIa* | 195 |
| 44-Kpn-17 | AZT, CFZ, FEP, CRO, CAZ, GEN, TOB, CIP, NIT, SXT | *bla*_CTX-M-15_, *bla*_TEM-1,_ *aac(6’)-Ib, aac(3’)-IIa* | 1 | 195 | 44-Tc | AZT, CFZ, FEP, CRO, CAZ, CIP, GEN, TOB | *bla*_CTX-M-15_, *bla*_TEM-1,_ *aac(6’)-Ib, aac(3’)-IIa* | 195 |
| 45-Kpn-17 | PTZ, AZT, CFZ, FEP, CRO, CAZ, CIP, GEN, TOB, NIT, SXT | *bla*_CTX-M-15_, *bla*_TEM-1,_ *aac(6’)-Ib, aac(3’)-IIa* | 1 | 195 | 45-Tc | PTZ, AZT, CFZ, FEP, CRO, CAZ, GEN, TOB, | *bla*_CTX-M-15_, *bla*_TEM-1,_ *aac(6’)-Ib, aac(3’)-IIa* | 195 |
| 49-Kpn-17 | AZT, CFZ, FEP, CRO, CAZ, CIP, AMK, GEN, TOB, NIT, SXT | *bla*_CTX-M-15_, *bla*_TEM-1,_ *aac(6’)-Ib, aac(3’)-IIa* | 1 | 195 | 49-Tc | AZT, CFZ, FEP, CRO, CAZ, GEN, TOB, | *bla*_CTX-M-15_, *bla*_TEM-1,_ *aac(6’)-Ib, aac(3’)-IIa* | 195 |
| 51-Kpn-17 | AZT, CFZ, FEP, CRO, CAZ, GEN, TOB, NIT, SXT, COL | *bla*_CTX-M-15_, *bla*_TEM-1,_ *aac(6’)-Ib, aac(3’)-IIa* | 1 | >195 | 51-Tc | AZT, CFZ, FEP, CRO, CAZ, GEN, TOB, | *bla*_CTX-M-15_, *bla*_TEM-1,_ *aac(6’)-Ib, aac(3’)-IIa* | >195\| |
| 53-Kpn-17 | FEP, CRO, GEN, TOB, SXT, COL | *bla*_CTX-M-15_, *bla*_TEM-1,_ *aac(6’)-Ib, aac(3’)-IIa* | 1 | 195 | 53-Tc | FEP, CRO, GEN, TOB | *bla*_CTX-M-15_, *bla*_TEM-1,_ *aac(6’)-Ib, aac(3’)-IIa* | 195 |
| 56-Kpn-17 | AZT, CFZ, FEP, CRO, CAZ, GEN, TOB, SXT | *bla*_CTX-M-15_, *bla*_TEM-1,_ *aac(6’)-Ib, aac(3’)-IIa* | 1 | 195 | 53-Tc | AZT, CFZ, FEP, CRO, CAZ, GEN, TOB | *bla*_CTX-M-15_, *bla*_TEM-1,_ *aac(6’)-Ib, aac(3’)-IIa* | 195 |
| 59-Kpn-17 | AZT, CFZ, FEP, CRO, CAZ, GEN, TOB, SXT | *bla*_CTX-M-15_, *bla*_TEM-1,_ *aac(6’)-Ib, aac(3’)-IIa* | 2 | 67  >195 | 59-Tc | AZT, CFZ, FEP, CRO, CAZ, GEN, TOB | *bla*_CTX-M-15_, *bla*_TEM-1,_ *aac(6’)-Ib, aac(3’)-IIa* | >195 |
| 61-Kpn-17 | AZT, CFZ, FEP, CRO, CAZ, GEN, TOB, CIP, NIT, SXT, COL | *bla*_CTX-M-15_, *bla*_TEM-1,_ *aac(6’)-Ib, aac(3’)-IIa* | 1 | 195 | 61-Tc | AZT, CFZ, FEP, CRO, CAZ, GEN, TOB | *bla*_CTX-M-15_, *bla*_TEM-1,_ *aac(6’)-Ib, aac(3’)-IIa* | 195 |
| 62-Kpn-17 | AZT, CFZ, FEP, CRO, GEN, TOB, NIT, SXT, COL | *bla*_CTX-M-15_, *bla*_TEM-1,_ *aac(6’)-Ib, aac(3’)-IIa* | 1 | >195 | 62-Tc | AZT, CFZ, FEP, CRO, GEN, TOB | *bla*_CTX-M-15_, *bla*_TEM-1,_ *aac(6’)-Ib, aac(3’)-IIa* | >195 |
| 63-Kpn-17 | AZT, CFZ, FEP, CRO, CAZ, GEN, NIT, SXT, COL | *bla*_CTX-M-15_, *bla*_TEM-1,_ *aac(6’)-Ib, aac(3’)-IIa* | 1 | >195 | 63-Tc | AZT, CFZ, FEP, CRO, CAZ, GEN | *bla*_CTX-M-15_, *bla*_TEM-1,_ *aac(6’)-Ib, aac(3’)-IIa* | >195 |
| 65-Kpn-17 | AZT, CFZ, CRO, CAZ, TOB, CIP, NIT, SXT | *bla*_CTX-M-15_, *bla*_TEM-1,_ *aac(6’)-Ib, aac(3’)-IIa* | 1 | >195 | 65-Tc | AZT, CFZ, CRO, CAZ, TOB | *bla*_CTX-M-15_, *bla*_TEM-1,_ *aac(6’)-Ib, aac(3’)-IIa* | >195 |
| 70-Kpn-17 | PTZ, AZT, CFZ, FEP, CRO, CAZ, GEN, TOB, CIP, NIT, SXT | *bla*_CTX-M-15_, *bla*_TEM-1,_ *aac(6’)-Ib, aac(3’)-IIa* | 2 | 100  195 | 70-Tc | PTZ, AZT, CFZ, FEP, CRO, CAZ, GEN, TOB | *bla*_CTX-M-15_, *bla*_TEM-1,_ *aac(6’)-Ib, aac(3’)-IIa* | 195 |
| 72-Kpn-17 | AZT, CFZ, FEP, CRO, CAZ, AMK, GEN, TOB, NIT, SXT | *bla*_CTX-M-15_, *bla*_TEM-1,_ *aac(6’)-Ib, aac(3’)-IIa* | 1 | >195 | 72-Tc | AZT, CFZ, FEP, CRO, CAZ, GEN, TOB, | *bla*_CTX-M-15_, *bla*_TEM-1,_ *aac(6’)-Ib, aac(3’)-IIa* | >195 |
| 75-Kpn-17 | AZT, CFZ, FEP, CRO, CAZ, GEN, TOB, CIP, NIT, SXT, COL | *bla*_CTX-M-15_, *bla*_TEM-1,_ *aac(6’)-Ib, aac(3’)-IIa* | 1 | 195 | 75-Tc | AZT, CFZ, FEP, CRO, CAZ, GEN, TOB | *bla*_CTX-M-15_, *bla*_TEM-1,_ *aac(6’)-Ib, aac(3’)-IIa* | 195 |
| 77-Kpn-17 | AZT, CFZ, CRO, CAZ, TOB, NIT, SXT,COL | *bla*_CTX-M-15_, *bla*_TEM-1,_ *aac(6’)-Ib,* | 1 | 195 | 77-Tc | AZT, CFZ, CRO, CAZ, TOB | *bla*_CTX-M-15_, *bla*_TEM-1,_ *aac(6’)-Ib* | 195 |
| 78-Kpn-17 | PTZ, AZT, CFZ, FEP, CRO, CAZ, GEN, TOB, CIP, SXT | *bla*_CTX-M-15_, *bla*_TEM-1,_ *aac(6’)-Ib, aac(3’)-IIa* | 1 | 195 | 78-Tc | PTZ, AZT, CFZ, FEP, CRO, CAZ, GEN, TOB | *bla*_CTX-M-15_, *bla*_TEM-1,_ *aac(6’)-Ib, aac(3’)-IIa* | 195 |
| 80-Kpn-17 | AZT, CFZ, FEP, CRO, CAZ, GEN, TOB, CIP, NIT, SXT, COL | *bla*_CTX-M-15_, *bla*_TEM-1,_ *aac(6’)-Ib, aac(3’)-IIa* | 2 | 86  >195 | 80-Tc | AZT, CFZ, FEP, CRO, CAZ, GEN, TOB | *bla*_CTX-M-15_, *bla*_TEM-1,_ *aac(6’)-Ib, aac(3’)-IIa* | >195 |

PTZ: piperacillin-tazobactam, AZT: Aztreonam, CFZ: cefazolin, FEP: cefepime, CAZ: ceftazidime, CRO: ceftriaxone, CIP: ciprofloxacin, AMK: amikacin, GEN: gentamicin, TOB; tobramycin, CIP: ciprofloxacin, NIT: nitrofurantoin, TGC: tigecycline, SXT: trimethoprim-sulfametoxazole, COL: colistin.
